# Single Fetal Demise in Twin Pregnancy—A Great Concern but Still a Favorable Outcome

**DOI:** 10.3390/diseases9020033

**Published:** 2021-04-29

**Authors:** Bogdan Ioan Stefanescu, Ana-Maria Adam, Georgiana Bianca Constantin, Constantin Trus

**Affiliations:** 1 Department of Obstetrics and Gynecology, Faculty of Medicine and Pharmacy, University ”Dunarea de Jos” Galati, 800008 Galati, Romania; adam.anamari89@gmail.com; 2 Department of Morphological Sciences, Faculty of Medicine and Pharmacy, University ”Dunarea de Jos” Galati, 800008 Galati, Romania; constantin_bianca2009@yahoo.com (G.B.C.); dr.bogdan.stefanescu@gmail.com (C.T.)

**Keywords:** twin pregnancy, single fetal demise, fetal loss, twin pregnancy complications

## Abstract

The incidence of multiple pregnancies has increased in the last decades, mostly explained by the more frequent use of ovulation induction drugs and assisted reproduction techniques. Although single fetal death in the first trimester of twin pregnancy is not an uncommon event nor does it have serious consequences on the survival fetus, the death of one fetus in the second or third trimester of pregnancy is associated with a serious increase in morbidity and mortality for the surviving co-twin. Preterm labor, preeclampsia, intrauterine growth restriction (IUGR), neurological complications or even the death of the surviving twin have been associated with single fetal demise after mid gestation. We present a very rare case of twin pregnancy with single fetal demise at 26 weeks of gestation successfully managed to term.

## 1. Introduction

Single fetal loss of a twin pregnancy during the first trimester is not an uncommon event and seems not to impair the further development of the survival one [1]. This situation is described in the literature as the “vanishing twin syndrome”. According to Landy et al. [2], the rate of disappearance could be as high as 29%.

In contrast, the death of a twin in the late second or third trimester of pregnancy is a rare obstetric complication associated with increased maternal and fetal morbidity and mortality. Apart from important psychological stress to both parents and attending obstetrician, this condition is highly associated with preterm labor, preeclampsia, intrauterine growth restriction (IUGR), neurological complications or even the death of the surviving twin, as well as maternal disseminated intravascular coagulation (DIC).

In the case of pregnancy continuation, the dead twin will progressively transform into “fetus papyraceous” due to the absorption of the soft tissues, placental and amniotic fluids. The dead fetus will be found compressed between the amniotic sac of the survival twin and the uterine wall [3].

## 2. Case Report

We present a case of a 24 years old pregnant woman, G2, P2, who was addressed to our department for a second trimester ultrasound scan.

She had one previous pregnancy 2 years before, complicated with late term preeclampsia. She did not receive prophylactic therapy with aspirin as none of the risk factors were identified at that moment. The male fetus was delivered by caesarean section at 38 weeks and 4 days of gestation. There was no history of twinning in her family, nor taking ovulation inducing drugs.

**Figure 1:**
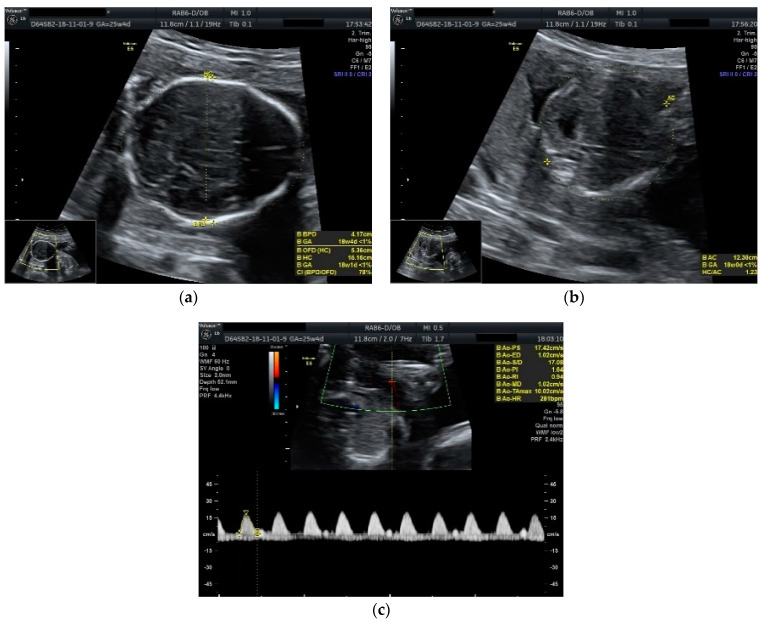
Ultrasound scan performed at 25 weeks and 4 days of gestation: (**a**) biparietal diameter of the growth restricted fetus appropriate for 18 weeks and 4 days of gestation; (**b**) abdominal circumference of the growth restricted fetus appropriate for 18 weeks and 0 days of gestation; (**c**) umbilical artery Doppler scan of the growth restricted fetus showing the absence of diastolic flow.

Obstetric ultrasonography showed a diamniotic dichorionic twin pregnancy, both fetuses alive, with discordant growth. Measurements for the first fetus were appropriate for 25 weeks and 4 days of gestation with normal amniotic fluid volume, whereas the biometry for the second one revealed much lower dimensions corresponding to 18 weeks and 4 days of gestation with low amniotic fluid volume. No fetal anomalies were seen. A Doppler scan showed increased pulsatility and resistivity indices in the umbilical artery for the growth restricted fetus (PI: 1.64, RI: 0.94, absent diastolic flow and sinusoidal pattern of the venous umbilical flow) (Figure 1). 

In these circumstances, counseling was offered regarding the potential unfavorable outcome for the pregnancy in general and especially for the second fetus, who already showed signs of distress. 

A second ultrasound scan, performed after 3 days, revealed a normal growing fetus appropriate for 26 weeks of gestation along with the second dead fetus. 

**Figure 2:**
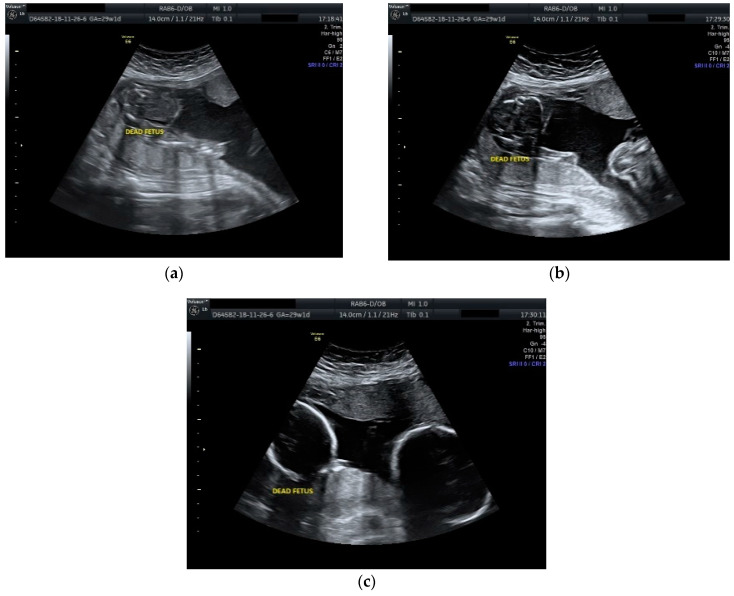
Ultrasound scan performed at 29 weeks and 1 day of gestation: (**a**), (**b**) dead fetus along with normal growing fetus with normal amniotic fluid; (**c**) cephalic extremities of the two fetuses with evident growth discordance.

**Figure 3:**
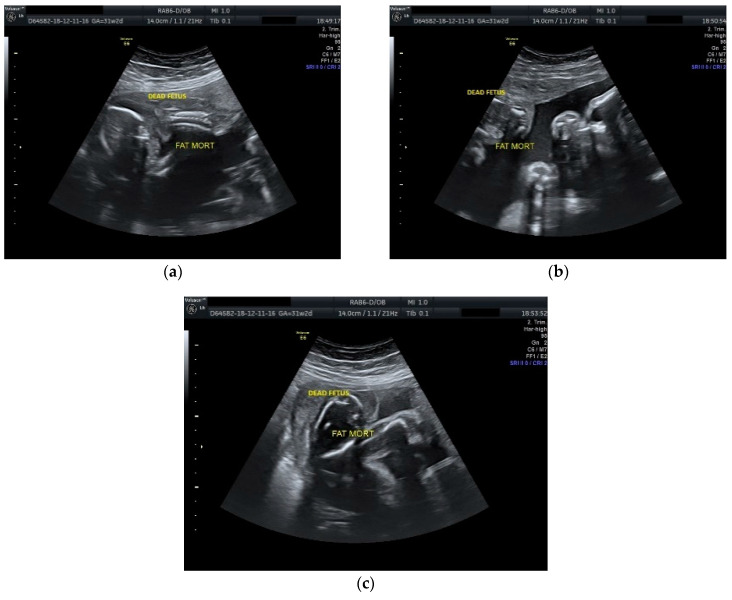
Ultrasound scan performed at 31 weeks and 2 days of gestation: (**a**), (**b**), (**c**) dead fetus with consistent plastic changes along with normal growing fetus with normal amniotic fluid.

The case was managed conservatively with regular monitoring of the maternal coagulation profile, along with intensive fetal surveillance for the surviving twin (Figure 2 and Figure 3).

Low-molecular-weight heparin in prophylactic dose was prescribed until the birth. Maternal monitoring included once every 2 weeks CBC and coagulation profile (PT, INR, APTT, and serum fibrinogen). All were within the normal limit until delivery. Fetal monitoring included an ultrasound scan every 2 weeks, and a nonstress test (NST), started at 32 weeks biweekly, which was all reactive.

Four doses of 6 mg dexamethasone each, 12 h apart, were given for lung maturation at 32 weeks of gestation.

**Figure 4:**
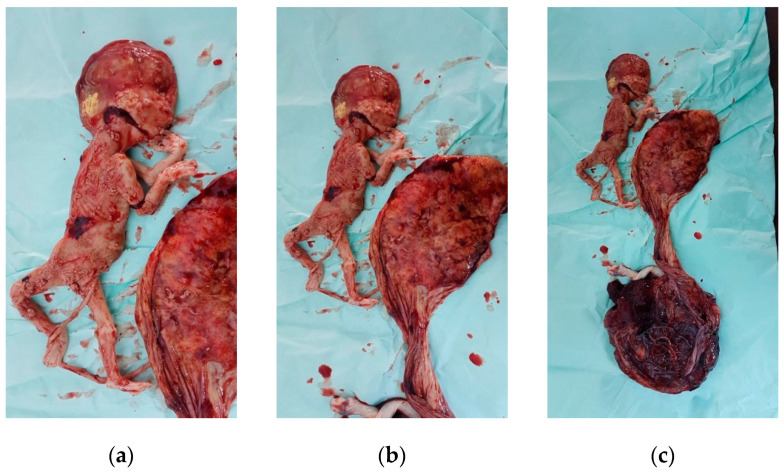
Macerated fetus: (**a**), (**b**) macerated fetus with the placenta; (**c**) macerated fetus with both placentae with evident different dimensions.

After consultation with a pediatrician and anesthesiologist, the patient delivered by cesarean section under spinal anesthesia at 38 weeks and 5 days of gestation a live female fetus weighted 3100 g Apgar score 8 at 1 min and 9 at 5 min with good adaptation, as well as a second macerated female fetus of approximately 400 g (Figure 4).

The dead fetus, with its very thin trivascular umbilical cord measuring approximately 20 cm in length and the placenta of 17 cm in diameter weighing 180 g were sent for autopsy, which showed no detectable cause for fetal demise.

The postpartum course was uneventful. Both the mother and the baby were discharged at the sixth postoperative day.

## 3. Results and Discussions

The incidence of single fetal death in twin pregnancies is reported to be as high as 2.5% to 6.0%, compared to 0.3% to 0.6% in singleton pregnancies [4,5]. According to Enbom, the incidence of twin pregnancies with single fetal demise ranges from 0.5% to 6.8% [6,7].

Intrauterine single fetal death can occur at any gestational age. If this event happens in the first trimester of the pregnancy, the surviving twin will most likely develop without further consequences. However, if the fetal death occurs after mid gestation (17 weeks’ gestation) there is an associated increased risk of preterm labor, IUGR, preeclampsia, and perinatal mortality [5,8]. In contrast, if the single fetal death occurs at 33 weeks of gestation or above, the other twin will have better chances of survival [1]. 

The incidence of single fetal demise is higher in monochorionic than in dichorionic pregnancies. Monochorionicity is reported in 50%–70% of twin pregnancies with intrauterine single fetal death [9,10]. In a study published by Woo et al. [3], 83% of cases (five out of six) were monochorionic pregnancies.

The causes of single fetal demise in a twin pregnancy are represented by twin–twin transfusion, placental insufficiency, placental abruption, IUGR related to pre-eclampsia, discordant growth, velamentous insertion of cord, cord stricture or true knot, cord around the neck, congenital abnormalities and blunt abdominal injury [2,11,12].

As stressed in numerous previous studies, chorionicity rather than zygosity determines the risk of complications in the surviving twin [13,14,15]. In a study published by Arinkan et al. [13], there was a 13 times greater risk for premature delivery for the surviving fetus, as well as a seven times greater risk for abruptio placentae in the monochorionic group compared with dichorionic group with single fetal demise. That is why the assessment of chorionicity by ultrasonography as early as possible in twin pregnancy is mandatory. 

The risk of mortality and morbidity in the surviving twin is three to four times greater in monochorionic than in dichorionic pregnancies. After Livnat et al. [16], the risk of co-twin death in monochorionic pregnancy is 12%, as compared with a 4% risk in dichorionic pregnancies. This could be explained by placental vascular anastomoses, which can be seen in up to 98% of monochorionic pregnancies [11]. Thus, the death of one fetus can lead to acute hypotension and multiorgan ischemia of the co-twin. Other mechanisms such as transchorionic embolization from the deceased twin or coagulation abnormalities have been suggested in the literature [3].

In addition, a wide range of structural abnormalities have been reported in the surviving twin such as neural tube defects, optic nerve hypoplasia, hypoxic ischemic lesions of the white matter (multicystic encephalomalacia), microcephaly (cerebral atrophy), hydranencephaly, porencephaly, hemorrhagic lesions of white matter, posthemorrhagic hydrocephalus, and bilateral renal cortical necrosis [10]. In a study published by Woo et al., one surviving fetus out of five monochorionic pregnancies with single fetal demise was diagnosed by ultrasonography with left frontoparietal infarction [3]. On the other hand, in diamniotic pregnancies, the death of one fetus could have no effect on the other twin. Other circumstances that could impair the survival twin could be maternal medical conditions or intrauterine infections responsible for the death of the first fetus.

The most important maternal complication following fetal demise is DIC. In rare cases, the release of fibrin and tissue thromboplastins from the dead fetus in the maternal circulation will activate the extrinsic coagulation pathway and subsequently induce DIC. 

Although potentially fatal for both mother and fetus, maternal coagulopathy appears to be uncommon [17]. According to different studies published by Romero et al. [18], Landy et al. [2], Pritchard and Ratnoff [19], the incidence of maternal DIC following single fetal demise will not exceed 25% of cases. In a study published by Tunc et al., none of the 29 cases of twin pregnancy complicated with single fetal demise developed DIC [20]. Moreover, coagulopathy has been reported to occur in about 3–5 weeks following fetal demise [1]. Therefore, when single fetal death occurs in twin pregnancy after the first trimester, an initial maternal clotting profile with reassessment in 2–3 weeks is reassuring.

Once the diagnosis of twin pregnancy with single fetal demise is established, the pressure for both the patient and the medical team is considerable. This is explained by the lack of certainty regarding all the potential complications for the mother and surviving fetus as well. In these circumstances, there is always a big dilemma on leaving the living fetus exposed to potentially distressing factors which killed the first twin in the first place or extracting it, knowing all the risks associated with prematurity. More studies are needed to clearly advise on proper management. Until then, conservative management until 37 weeks of gestation is advocated by most of the authors in the absence of other obstetric indications.

Intensive fetal monitoring is mandatory and includes frequent non-stress testing, biophysical profiling, ultrasound scans and umbilical artery Doppler velocimetry in order to evaluate fetal growth and condition, possible associated fetal anomalies as well as amniotic fluid volume. 

Woo et al. presented in their paper some key points in the management of twin pregnancies with single fetal demise (Table 1).

In most of the twin pregnancies complicated with single fetal demise, the labor will spontaneously develop within the next weeks. After D’Alton et al. [21], approximately 90% of cases will deliver within 3 weeks after fetal death. Yet, in other different studies from the literature, the reported median interval between single fetal death and delivery of the second twin was 5 weeks [1,7], 7 weeks [22] or 9 weeks [11]. In our case, the pregnancy successfully continued for almost 12 weeks, being one of the longest ever reported.

Despite the fact that single fetal death in twin pregnancy alone is not an indication for caesarian section, this mode of delivery was the most frequently reported in the literature. Nevertheless, there are a lot of additional factors that need to be considered in order to recommend the safest way of delivery for both the mother and the surviving fetus. Thus, gestational age, presentation and biological condition of the surviving twin, location of the dead fetus in the uterine cavity that can obstruct the birth canal, other obstetrical conditions such as placenta previa or abruptio placentae, coagulation profile or other maternal medical conditions should be carefully evaluated. Due to a previous birth by cesarean section, we decided to surgically manage the case once the labor begun.

Although it is recommended that after delivery, both the dead fetus and the placenta should be pathologically examined, in most cases, this will not offer any clue at all about the possible cause of fetal death. This is explained by the advanced state of tissue autolysis of the demised fetus and placenta at the moment of delivery [9]. 

The overall prognosis is poor for monochorionic pregnancies with single fetal death and is mostly explained by the increased morbidity and mortality of the surviving fetus. In dichorionic twins, the prognosis for the surviving twin is relatively good and immaturity is the main risk factor.

## 4. Conclusions

The prognosis and outcome of a single fetal death in a twin pregnancy is very much dependent on the gestational age at the moment of diagnosis and placentation. That is why the determination of chorionicity should be carried out very early in the pregnancy. In case of diamniotic dichorionic twin pregnancy with single fetal death, with good surveillance, the live fetus can be salvaged. In our case, successful prolongation of pregnancy could be maintained up to 38 weeks and 5 days.

Yet, the absence of large-scale studies makes it difficult to advise the parents on the prognosis and optimal treatment, but conservative management is preferred by most obstetricians.

## Figures and Tables

**Table 1:** Key points in management of twin pregnancies with single fetal demise (adapted from [3]).

1. Counseling and support.
2. Individualized management plan.
3. Management in a tertiary center with competent neonatal support.
4. Information on chorionicity.
5. Evaluation of fetal anomalies and close fetal surveillance.
6. Steroid prophylaxis for lung maturity in case of preterm delivery.
7. Conservative management until 37 weeks. Earlier intervention in the presence of other obstetric indications.
8. Vaginal delivery if possible.
9. Post-mortem examination of the stillborn. Placenta for histological examination.
10. Pediatric assessment and long-term follow-up.

## Data Availability

Not applicable.
